# Supplementary material for: SARS-CoV-2 antibody prevalence in a pediatric cohort of unvaccinated children in Mérida, Yucatán, México
Source: PLOS Glob Public Health. 2022 Jun 15;2(6):e0000354. doi: 10.1371/journal.pgph.0000354 (PMC10021704; doi:10.1371/journal.pgph.0000354)
Supplement: Attachment — Submitted filename: 031722 Response to Reviewers.docx [file pgph.0000354.s002.docx]

Please find below our response to reviewer’s comments, in red below the comment. Thanks to all reviewers, we believe their help has significantly improved the manuscript.

**Journal Comments**

1. Please include a complete copy of PLOS’ questionnaire on inclusivity in global research in your revised manuscript. Our policy for research in this area aims to improve transparency in the reporting of research performed outside of researchers’ own country or community. The policy applies to researchers who have travelled to a different country to conduct research, research with Indigenous populations or their lands, and research on cultural artefacts. The questionnaire can also be requested at the journal’s discretion for any other submissions, even if these conditions are not met.  Please find more information on the policy and a link to download a blank copy of the questionnaire here: https://journals.plos.org/plosone/s/best-practices-in-research-reporting. Please upload a completed version of your questionnaire as Supporting Information when you resubmit your manuscript.

This information has been provided along with the revised manuscript.

2. In the online submission form, you indicated that "All data that support the findings of this work are available, upon request, from JTE.". All PLOS journals now require all data underlying the findings described in their manuscript to be freely available to other researchers, either 1. In a public repository, 2. Within the manuscript itself, or 3. Uploaded as supplementary information.
This policy applies to all data except where public deposition would breach compliance with the protocol approved by your research ethics board. If your data cannot be made publicly available for ethical or legal reasons (e.g., public availability would compromise patient privacy), please explain your reasons by return email and your exemption request will be escalated to the editor for approval. Your exemption request will be handled independently and will not hold up the peer review process but will need to be resolved should your manuscript be accepted for publication. One of the Editorial team will then be in touch if there are any issues.

The data and a link to it (doi:10.17632/h43mdmz6vv.1) are now included in the methods section (line 161). Access to the data will be embargoed until acceptance of the manuscript.

3. Please provide us with a direct link to the base layer of the map used in Figs 1 and 4 and ensure this location is also included in the figure legend.

The figure legends of Figs 1 and 4 now includes the following statement: “Base layers were downloaded from Mexico’s census database (https://www.inegi.org.mx/app/mapa/espacioydatos/default.aspx).”

**Reviewer Comments**

Reviewer #1: I indicated "No" in response to the question "Has the statistical analysis been performed appropriately and rigorously". This is not because the analyses that the authors conducted are flawed, but because there are three testable factors (that I can identify) which could have led to spurious conclusions, but which were not tested. Once the authors explore these questions, the statistical analysis should be deemed appropriate and rigorous:

1. Is there any correlation between the arm of the RCT in which children are enrolled and SARS-CoV-2 antibody prevalence? Could this have any impact on the conclusions?

The authors do not see this as a source of bias in the study. We are considered the entire study population and, thus, a combination of both arms of the trial. Since we are testing a large number of samples distributed throughout the city, we believe there is no value in linking SARS-CoV-2 seroprevalence to the arm children were enrolled to. While such analysis could be done, there is no epidemiological value of it to the description of population-level antibody seroprevalence to SARS-CoV-2.

2. What are the rates of non-response or non-consent to the socio-demographic survey and blood draws? Could this have any impact on the conclusions?

This analysis was done on a pool of banked blood specimens from individuals who consented to the TIRS trial and that allowed their specimens for ‘future use’. As such, there is no value in reporting non-responses because they were not related to the purpose of SARS-CoV-2 testing of the specimens. We also considered individuals with complete data (blood specimen and demographic survey) to characterize the population-level antibody response to SARS-CoV2 in children.

3. Given the rapidly-changing incidence rates and relatively large wave of infection that happened in Mexico in January of 2021, I assume that the clusters which were surveyed later in the study have higher seroprevalence than the clusters which were surveyed early, unless this study somehow surveyed households as a simple random sample. Please: a) Confirm whether the survey protocol sampled whole clusters in serial or whether the survey protocol was able to approximate a random sample of households over time, and b) Report whether/how the statistical analyses (the age/sex risk factor analysis, the household size correlation analysis and the K-function spatial analysis) accounted for temporal confounding in drawing their conclusions. For example, could it be possible that the spatial analysis found no tendency for neighboring clusters to share high seroprevalence because of the order in which the clusters were sampled? Could it be possible that household size was found to correlate with seroprevalence because clusters tended to have larger household size later in the surveying protocol?

We agree that SARS-CoV2 infection dynamics have changed rapidly. However, we believe this did not affect our results, since we aimed for a relatively “short” cross-sectional study rather than a longitudinal characterization of antibody prevalence to SARS-CoV-2. We collected 85% of the 1005 specimens within a 62-day period (Feb4-Apr 6), with 143 specimens collected in May and early June. Specifically, answering your questions: a) the general specimen collection protocol involved visiting multiple clusters at the same time in a quasi-random fashion. We sampled clusters at different times and re-visited clusters to collect samples from children who were not available for the original sampling. We are attaching a reviewer only figure showing that rates of incidence do not increase significantly over this short time. b) given (a), we did not conduct any temporal analysis of our dataset. We consider the time window was too short and the sample size too imbalanced to explore changes in spatial pattern over time. C) the study was conducted in an area that is demographically similar, so we do not have any indication that household size would differ dramatically across clusters.

In addition, I have three suggestions that I believe will enhance the quality and significance of the manuscript:

1. Please include the complete socio-demographic survey instrument used in this study, if not restricted by original IRB. Why were age, sex, and household size the only determinants explored?

We considered age, sex, and household size as the variables that might address our questions. Additionally, most of the sociodemographic variables we have collected are related to the Arbovirus infection and mosquito bionomics and dynamics not relevant for this study. We are including a copy of the survey instrument in our Mendeley data repository for the study (doi:10.17632/h43mdmz6vv.1).

2. What is the justification for testing a binary variable (>5 inhabitants vs <=5 inhabitant) instead of a continuous household size variable? The linear odds ratio presented in Table 2 seems sufficient.

Given that one of the main predictors of SARS-CoV2 seroprevalence was household size, we evaluated this metric using two different (but related) indices. We used the linear model to test for the association with HH size as a continuous variable. Additionally, we used a binary variable (HH size lower or higher than 5) as a measure of crowding rather than plain household size. Interestingly, both predictors show association with SARS-CoV-2, though it appears that the trend in the linear association may be driven by the higher seroprevalence found in crowded houses. As such, we consider both analyses to be important to the conclusions of the study.

3. I believe there is a discrepancy in the definition of a large household size as described in the Methods and Results sections. The Methods section states "houses with more than five inhabitants" and the Results section states "homes with more than six inhabitants". Were two definitions tested or should one of these sentences be corrected?

The text in the results section (line 289) has been changed to reflect the correct definition.

Reviewer #2: Overall comment
The authors have touched upon a very important topic. There is limited evidence about pediatric infection with COVID-19 relative to the massive evidence that has been generated about the disease in adults since the onset of the pandemic. The authors have also used very robust statistical and analytical techniques to analyze the data and have presented it well.

We greatly appreciate the kind words of reviewer #2 and thank her or him for their assistance.

Introduction
The ending paragraph of the introduction need not carry the details of the methodology or study setting as the details are mentioned in the methods section.

The authors believe that the methodology and study setting is an important factor for the significance of the work. We have shortened, but not removed, this part of the introduction (lines 86-96).

Methods
Study design and sample size
While the authors have done well to mention that the samples that were analyzed for the study were part of a larger on-going trial and were randomly selected, the method followed for random selection of samples has not been specified.
If the original study was using a cluster randomization, how was this taken into account when selecting the samples for this study?

The study aimed to test samples for SARS-CoV2 antibodies during the spring of 2021 as a requisite for our cross-sectional study design. As such, we did not conduct a random selection of samples, but tested the majority of samples collected for the cohort.

What were the parameters or underlying assumptions for the sample size?

We tested all specimens that met the eligibility criteria (see below) and were collected in the spring of 2021.

The eligibility criteria for selection of participants have not been stated clearly either. Though it is mentioned that none of the participants had a prior reported COVID-19 infection, it is unclear if that had any basis for study eligibility? Lines 85-86 (page 4; Introduction) and lines 121-122 (Page 5; Methods) seem like contradictory statements and should be revised. If the aim of the study was to estimate the prevalence of COVID-19 among children through measurement of antibodies, then excluding those with a history of prior infection would lead to an underestimation.

The criteria did not involve a history of SARS-CoV-2 exposure as no data was taken in that regard. The text stating that has been corrected. Our criteria included: samples being collected during the spring of 2021; individuals providing a demographic survey; participants provided a ‘future use’ approval for using their samples; participants were enrolled in the TIRS trial.

Also, the participants of this study were part of a trial and may have been selected through an eligibility criteria that set them apart from the general population. How would this bias the result or what impact would it have on the estimates?

The eligibility criteria of the trial depend on living in a randomized neighborhood cluster within the city of Merida. All eligible children in these clusters were asked to participate in the trial and all children who consented were included in this serosurvey. Thus, we believe the samples are representative of the population in general. We have- corrected a statement in the results section (lines 201-202) and added another (lines 202-205) to make this more clear.

Also, if more than one eligible child was present in the household, were all selected?

Yes. We tested all children with a blood specimen within a month including those who were co-habitating. This has been added to the methods (line 132-133)

A lot of repetition about the enrollment and sample collection throughout the text. It is unclear what information was collected at the time of enrollment. There was a considerable lag between enrollment period and period of sample collection. Was the information on prior history of COVID-19 collected at the time of enrollment or time of sample collection?
How long after collection were the samples analyzed for the presence of anti-SARS-CoV-2 antibodies?

We agree and thank the reviewer for pointing this out. Information about SARS-CoV-2 exposure was not collected at time of blood sampling, this text in the manuscript has been corrected. Testing was performed soon after collection which is now reflected in the text (lines 129-130). The samples were tested for anti-SARS-CoV-2 antibodies 2-3 months after collection because we ran tests for detecting IgG antibodies against Aedes-borne viruses first, per the requirements of the trial.

Analysis
The abstract states that the socioeconomic and demographic factors were also studied. Apart from household size, there is no mention of any other socioeconomic measure in the analysis. If other measures were examined and no difference was observed, it should be mentioned in the results.

A statement that all factors except household size had no effect has been added to the results section (line 272-273).

The authors could have also collected information regarding crowding index that takes into account the number of living spaces in the house/dwelling for the given number of occupants. That may be a better predictor of household transmission than just the household size. It also gives an estimate of any difference due to socio-economic status.

We did collect other demographical data, such as number of living spaces in the house/dwelling for the given number of occupants (as the reviewer is describing). We have tested these variables but found no significant effects. These variables are further complicated by the fact that the number of rooms in an average house in Mérida is not a reliable predictor of how crowded a given house is as often living rooms and other rooms are also used as bedrooms. Due to these factors, we determined it was best to report strictly the number of inhabitants per house.

Table 2 footnote should also indicate the reference category used for household size

This statement has been added (Table 2, legend)

Results
There is too much discussion in the results section. Some details mentioned in the results would be more appropriate in the discussion section.

We have removed some text in the results to avoid too much discussion.

Discussion
Study strengths and limitations have not been discussed. It would be helpful to know the possible impact that they can have on the estimates obtained in the study.

We have added statements in the discussion about study strengths and weaknesses (lines 360-365)

Reviewer #3: It is a study that provides valuable information to understand the dynamics of SARS-CoV-2 transmission in children.

The authors thank reviewer #3 for their kind words.

However, it is necessary to clarify the methodology, beginning with the study's design. The authors declare it is a cohort study, but it seems that there is no follow-up. It looks like a cross-sectional study nested in a longitudinal design (the TIRS trial); please clarify and follow the STROBE statement to describe the central aspect of the methods.

We apologize if our description was not clear. The study design is indeed cross-sectional, it aimed at characterizing SARS-CoV2 antibodies in a pediatric population within the spring of 2020. Part of the confusion may have emerged from our intention to describe the TIRS trial and how our cohort was linked to it. To facilitate the interpretation of our study, we shortened the description of the TIRS trial and kept only the aspects related to this SARS-CoV2 antibody seroprevalence characterization. We have also confirmed the manuscript to follow STROBE guidelines.

It is unclear how the authors accessed the children and their blood samples. In line 118, they say, "We selected a subset of 1,005 serum samples of children for anti- SARS-CoV-2 antibody testing". But in line 125, they explain, "Field personnel collected blood specimens by house-to-house visits." These two phrases seem contradictory.

The serum samples that were used in this study came from participants of the TIRS trial who agreed to future use of blood specimens provided for that trial. Line 125 (now line 137) describes how these samples were obtained for the study and line 118 (now lines 127-128) describes simply that all the samples made available by consent were used for this study. We have amended lines 127-131 to make this more clear.

It is unclear if they included all the children who participated in the Targeted Indoor Residual Spraying (TIRS) trial in Mérida, and how that study enrolled the children. Were there rejections? It is unusual not to have it when requesting venipuncture for children.

We only tested specimens from children that consented to be part of the study and that provided a blood specimen during roughly the spring of 2021 (late February to June). The TIRS trial experienced many rejections for participation, but those played no role in this cross-sectional study.

Please provide the rational to include subjects who reported no previous or current symptoms of SARS-CoV-2 infection.

As mentioned above, this is a cross-sectional study of a population of children to determine how common exposure to SARS-CoV-2 is in a pediatric population. We included all children in our study design in order to get the most accurate results.

Finally, this is not the first Latin American study to include children, although it is probably the first with young children.

This statement has been removed.

Reviewer #4: I do appreciate Guadalupe et al… work. The authors have conducted large analysis incorporating from several samples.

Thank you for your kind comments.

However, corrections on introduction, Methods, result and Conclusion should be addressed well. In addition to this, grammar, editing and following the guideline on the tables and figures need to be addressed.

Abstract section
On the last part, it be better if you recommend based on your pertinent finding instead of saying "This is the first analysis of a large cohort of children in a Latin American country and the results may inform public health decisions …………" which seems like more general.

The statement referenced has been removed from the manuscript. We have added a sentence to the abstract to discuss a recommended course of future action (lines 49-51)

List the abbreviations of SARS, IgG and IgM (when you use them in the first time)

These abbreviations have been defined (lines 56-57 and 91)

Introduction
Paragraph one of this section is very interesting, but the line 54 of the paragraph one be best if you begin with definition of your outcome variable.

The outcome variable is defined in the last paragraph of the introduction as “the presence of either immunoglobulin G or M (IgG or IgM) antibodies specific for a recombinant SARS-CoV-2 spike protein” (lines 90-92)

Line 70: said that "Here, we provide insight on the prevalence…….." avoid the unnecessary words such us we, I and etc. throughout in the document.

We have reduced the use of pronouns in the text

The last paragraph of the introduction section [from line 81-88] should rewrite it again. This is better to be in the result and the methods section.

This paragraph has undergone extensive revision, but the authors believe the information is relevant to understanding the results and should stay in the introduction

General comment: The introduction is good but it need to address the severity, consequences and clear gap of the problem in this section.

We believe that these issues are addressed throughout the introduction. If there are specific problems with what is written, we will be happy to revise.

Method section
The study site is not clearly addressed for readers (the full address of the Mérida)

We have added the GPS coordinates of Merida’s center to the methods section (lines 112-113)

Data collection procedure, sample size determination, study populations, exclusion criteria's, data collectors, Measurements, data quality controls Need to be addressed well in this section.

We have revised the methods to more effectively describe these issues

Result Section
The narrative of the biographic data in this section need to address clearly

We do not understand what this comment is asking for, but we have thoroughly edited and reviewed the results and believe we have improved it over the previous version of the manuscript

Follow the guide lines of the Journal for the all the tables and figures.

We have conformed the tables and figures for the journal

Discussion
In the paragraph two (line 306) you discussed the finding of the study from other but the study areas of the other studies are not listed (even though it is cited as 13 and 14).

We have specified the study cites of these reports (lines 332-333)

There is no conclusion, limitation (optional) and Recommendation in this paper. It is better to include conclusion (inclusively recommendation under this).

The discussion and conclusions sections have been combined in this manuscript per the journal’s allowance “a mixed Discussion/Conclusions section (commonly labeled “Discussion”)”

Funding issue, Abbreviation and Acronyms are not incorporated in this paper

The authors are not aware of another requirement to report funding outside of the Financial Disclosure Statement. We have removed funding information from the acknowledgments per the journal’s instructions. The authors are not aware of a requirement from the journal of including am abbreviations and/or acronyms list. If these are required by the journal, we will gladly provide it.
